# Dietary Energy Density Affects Fat Mass in Early Adolescence and Is Not Modified by *FTO* Variants

**DOI:** 10.1371/journal.pone.0004594

**Published:** 2009-03-04

**Authors:** Laura Johnson, Cornelia H. M. van Jaarsveld, Pauline M. Emmett, Imogen S. Rogers, Andy R. Ness, Andrew T. Hattersley, Nicholas J. Timpson, George Davey Smith, Susan A. Jebb

**Affiliations:** 1 Cancer Research UK Health Behaviour Research Centre, Department of Epidemiology and Public Health, University College London, London, United Kingdom; 2 Department of Community Based Medicine, Bristol University, Bristol, United Kingdom; 3 School of Pharmacy and Biomolecular Sciences, University of Brighton, Brighton, United Kingdom; 4 Genetics of Complex Traits, Institute of Biomedical and Clinical Science, Peninsula Medical School, Exeter, United Kingdom; 5 Diabetes Genetics, Institute of Biomedical and Clinical Science, Peninsula Medical School, Exeter, United Kingdom; 6 Department of Oral and Dental Science, Bristol University, Bristol, United Kingdom; 7 MRC CAiTE Centre, Department of Social Medicine, Bristol University, Bristol, United Kingdom; 8 MRC Human Nutrition Research, Cambridge, United Kingdom; London School of Hygiene & Tropical Medicine, United Kingdom

## Abstract

**Background:**

Dietary energy density (DED) does not have a simple linear relationship to fat mass in children, which suggests that some children are more susceptible than others to the effects of DED. Children with the *FTO* (rs9939609) variant that increases the risk of obesity may have a higher susceptibility to the effects of DED because their internal appetite control system is compromised. We tested the relationship between DED and fat mass in early adolescence and its interaction with *FTO* variants.

**Methods and Findings:**

We carried out a prospective analysis on 2,275 children enrolled in the Avon Longitudinal Study of Parents and Children (ALSPAC). Diet was assessed at age 10 y using 3-day diet diaries. DED (kJ/g) was calculated excluding drinks. Children were genotyped for the *FTO* (rs9939609) variant. Fat mass was estimated at age 13 y using the Lunar Prodigy Dual-energy X-ray Absorptiometry scanner. There was no evidence of interaction between DED at age 10 y and the high risk A allele of the *FTO* gene in relation to fat mass at age 13 y (β = 0.005, p = 0.51), suggesting that the *FTO* gene has no effect on the relation between DED at 10 y and fat mass at 13 y. When DED at 10 y and the A allele of *FTO* were in the same model they were independently related to fat mass at 13 y. Each A allele of *FTO* was associated with 0.35±0.13 kg more fat mass at 13 y and each 1 kJ/g DED at 10 y was associated with 0.16±0.06 kg more fat mass at age 13 y, after controlling for misreporting of energy intake, gender, puberty, overweight status at 10 y, maternal education, TV watching, and physical activity.

**Conclusions:**

This study reveals the multi-factorial origin of obesity and indicates that although *FTO* may put some children at greater risk of obesity, encouraging a low dietary energy density may be an effective strategy to help all children avoid excessive fat gain.

## Introduction

Overweight and obesity currently affects 32% of children aged 10–11 y in England [1]. Childhood obesity is associated with serious consequences to health in both the short and long term [2]. The recent rise in obesity prevalence is primarily caused by environmental changes affecting diet and activity levels; however large variation in the prevalence of obesity within populations suggests that some children are more susceptible than others to the obesogenic environment.

Dietary energy density (DED) refers to the amount of energy consumed per unit weight of food. Compared to a low DED, a high DED will increase energy intake (EI) and raise the risk of weight gain if there is no decrease in the amount of food eaten. Highly controlled experimental studies have shown that adults tend to consume a constant weight of food, despite changes in energy density, thus an increase in energy density leads to higher EI, a concept referred to as passive overconsumption [3]. It is possible that the relationship between DED and EI may be a result of a correlation with high fat intake because fat is a key determinant of energy density. However, two studies [4], [5] that manipulated fat content while keeping DED constant demonstrated the effect of DED is independent of fat intake because EI was greater as %fat increased only when DED was allowed to vary as well, whereas when DED was held constant, changes in %fat had no effect on EI.

Water is also a major determinant of DED. Water contributes more to the weight of foods than any macronutrient, thus energy-dense foods are not necessarily those high in fat, but those that are dry [6]. For example a dry food like breakfast cereal (e.g. sugar coated cornflakes) and a high fat food like cheese (e.g. Cheddar) have the same energy density of 16.2 kJ/g [7]. Importantly DED encompasses all foods likely to lead to an increase in EI and weight gain, not just high fat foods and so may represent an obesogenic diet better than fat intake alone.

In free-living humans the effect of energy density may be reduced as people may choose to combine energy dense foods with less energy dense foods or may reduce their portion sizes of more energy dense food when they know its energy content [8]. A longer experiment lasting 2 weeks, where the energy density of all foods were manipulated, demonstrated that the increase in EI associated with high DED was maintained over 2 weeks and translated into short term body weight gain [9]. Furthermore longitudinal observational studies of free-living adults tend to support an effect of high DED on weight gain. One study found no evidence to support an effect on weight gain in a Danish adult population [10]. However, three other studies of US women found evidence for an association between higher DED and greater weight gain during pregnancy [11] or after 6 [12] or 8 years of follow-up [13].

In contrast, the effect of DED on fatness in children is not straightforward. A review of experimental studies in 1997 found that children tend to be more sensitive to changes in energy density and are much more likely than adults to reduce their consumption at a subsequent meal to compensate for a high energy density at a previous meal [14]. More recent experimental work suggests that the sensitivity that children have to changes in energy density tends to reduce with age [14], [15], [16]. Further support for a decline in sensitivity to innate appetite control can be found in an observational study of 220 free-living twins followed from age 4 to 11 years, which showed that a psychometric measure of satiety responsiveness declined with age [17]. Based on this there may not be a relationship between DED and fatness in young children, but a relationship may emerge in older children. There have been 2 prospective observational studies of DED and fatness in children. A small study in Northern Ireland of children selected for low and high risk of obesity based on parental weight status, found that each 1 kJ/g DED at age 7 y was associated with a 90% increase in the risk of being in the top tertile of fat gain between age 7 and 15 y (defined by change in fat mass index), but no linear trend was reported [18]. Analyses of data from a sub-sample of the Avon Longitudinal Study of Parents and Children (ALSPAC) found a non-linear trend so that each 1 kJ/g DED at age 7 y was associated with a 36% increase in the risk of excess adiposity (defined at as the top quintile of fat mass index) at age 9 y, although there was no evidence of a similar effect of DED at 5 y on excess adiposity at 9 y [19].

There is currently no evidence to support an effect of DED in young children (<7 y). It is possible that young children are not susceptible to the effects of increased DED because they have an enhanced ability to respond to internal appetite cues compared to older children and thus reduce their intake to compensate for an increased energy density. Furthermore, there is no evidence of a linear effect of DED on later fatness i.e. on a group level children with the most fat or fat gain tend to have eaten more energy dense diets but there is no uniform increase in fat mass associated with each kJ/g rise in DED. The absence of a linear trend suggests that some children are more susceptible to the effects of DED than others, which may be explained by the presence of an interaction with some other factor that alters susceptibility to increased EI in the presence of high DED.

A variant (rs9939609, A allele) of *FTO* has been associated with higher BMI in both adults and children as young as 7 y, which equated to a ∼3 kg difference in body weight between homozygous high and low risk allele carriers [20]. Further analysis of fat and non-fat mass separately found that the association of the high-risk A allele with weight was mainly attributable to changes in fat mass with a 14% difference across the three genotype groups compared to a 1% difference in non-fat mass across groups. The functional relevance of *FTO* to obesity is currently under investigation. To date gene expression studies have shown that *FTO* is highly expressed in the appetite control regions of the hypothalamus [21], [22]. The implication of *FTO* gene expression in regions of the hypothalamus associated with appetite control suggests that compromised control of energy intake may be the reason for excess weight gain in people carrying the high risk A allele. Evidence of decreased responsiveness to satiety, a psychometric measure of appetite control, has recently been observed in 8–11 year old twins carrying the high risk A allele [23]. Further evidence that appetite is compromised has been shown in ALSPAC where carriers of the high risk A allele had higher energy intake, which was independent of their larger body size [24].

If variation in *FTO* represents a genetic susceptibility to compromised appetite control, then it may modify the effect of exposure to a high DED on fat mass such that children without the high risk *FTO* allele are able to adjust their portion sizes to compensate for a higher DED. We assessed whether *FTO* variants modified the effect of DED at age 10 y on fat mass at age 13 y in the ALSPAC cohort.

## Methods

### Sample

**Figure 1:**
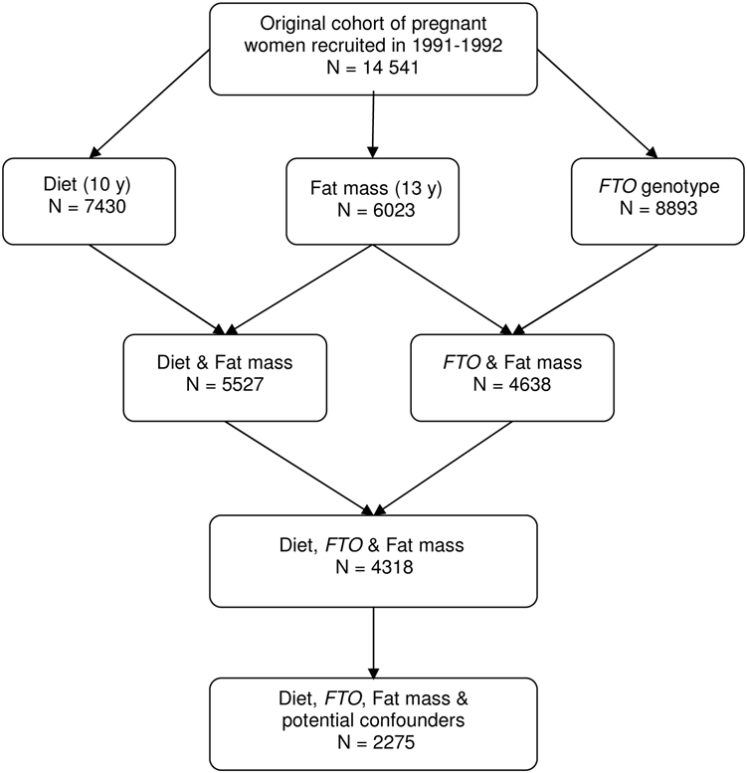
Diagram indicating numbers of children in the ALSPAC cohort with data available.

Data are from ALSPAC, a prospective cohort study, set up in 1991, to assess all aspects of pregnancy, infancy and childhood growth and development. A detailed account of study methodology can be found elsewhere [25]. Briefly, all pregnant women in Avon with an expected date of delivery between 1^st^ April 1991 and 31^st^ December 1992 were eligible for recruitment. A total of 14,541 pregnant women were enrolled in the study. For this analysis we have used data on *FTO* genotype, diet at age 10 y and body composition at age 13 y, which was available in varying numbers of children (**figure 1**). Data on genotype, diet and fat mass was available in 4318 children. Complete data on all variables, including potential confounders, was available in 2275 children. We certify that all applicable institutional and governmental regulations concerning the ethical use of human volunteers were followed during this research. Parents provided informed written consent for their child and ethical approval for the study was obtained from the ALSPAC Law and Ethics committee and local research ethics committees.

### Genetic data

Genotyping of rs9939609 was successful in 8,893 children. Genotyping was performed by KBiosciences (Hoddesdon, UK) using their own system of fluorescence based competitive allele-specific PCR (KASPar). Details of assay design are available from the KBiosciences website (http://www.kbioscience.co.uk).

### Dietary data

Dietary data was collected using 3-day unweighed diet diaries when children were aged 10.7±0.3 y. Children completed the diary, with parental help, before bringing it to a clinic. Diet recordings were requested to cover two weekdays and one weekend day. At clinics a nutritionist checked the diaries with the child (accompanied by a parent) and missing or ambiguous details were clarified to improve accuracy and completeness. If a diary had not been completed by the child a single 24 hour recall was administered at the clinic, this occurred in 13.5% of cases. Diet data was coded using DIDO software [26] and nutrient data was generated with an in house program using food composition information from the 5^th^ edition of McCance and Widdowson's food tables and supplements [7]. Portion sizes were coded using household measures and when these were missing a standard age 10 year old child's portion size was used based on data from the 1997 UK National Diet and Nutrition Survey [27]. Dietary energy density (kJ/g) (DED) was calculated (excluding drinks) by dividing total food energy (kJ) by total food weight (g). Drinks were excluded because experimental evidence for the effect of DED on energy intake is based on the manipulation of food composition, not the inclusion of extra drinks into the diet [28]. Drinks were defined as milk, fruit juice, fruit squash and cordials, fizzy drinks, water and flavoured water, hot chocolate, teas, coffee and alcohol. Daily energy intake (kJ/day) from all drinks was calculated and used as a covariate in regression analyses.

Misreporting of energy intake was estimated using an individualised method by calculating the ratio of reported energy intake (EI) to estimated energy requirements (EER) [29]. Individual EER were calculated using standard sex-specific equations based on body weight and an age appropriate coefficient for the energy needs of growth [30]. Physical activity was not assessed during the dietary recording period so no adjustment to EER was made for activity level. A 95% confidence interval for the accuracy of EI/EER was calculated. This 95% CI takes into account the level of precision that can be expected from the methods used to estimate EI and EER [31]. This 95% CI for EI/EER was 0.78–1.22. Therefore reports of EI between 78% and 122% of EER were considered to be plausible given the known limits of precision associated with estimating EI and EER and were defined as plausible reports (63% of the sample). Any children with an EI/EER<0.78 were defined as under-reporters (34% of the sample) and children with an EI/EER>1.22 were defined as over-reporters (3% of the sample). A categorical misreporting variable (under-, plausible-, and over-reporter) was used as a covariate in regression analyses.

### Fat mass and fat mass index

Fat Mass (kg) was measured by Dual-energy X-ray Absorptiometry, using the Lunar Prodigy fan beam scanner (GE medical systems, Chalfont St Giles, UK), when children were aged 12.8±0.2 y. Fat Mass Index (FMI) was calculated by dividing fat mass (kg) by height (m^1.3^) in order to adjust for body size. The optimal power to raise height to (1.3) was derived from the data so that the relationship between fat mass and height was completely removed [32].

### Potential confounders

Potential confounders were selected based on a literature search for variables that may be related to the exposures (*FTO* or DED) and the outcome (fat mass), but were not on the causal pathway. Where data on such variables was available they were included in adjusted analyses.

Having a large amount of fat mass is one of the biggest risk factors for having a high fat mass in the future. If a dietary factor is related to fatness at follow-up but is also related to fatness at baseline then causal inference may not be appropriate as the original high fatness (which may have been caused by some other factor) may simply be tracking. In order to separate a diet associated with the development of high fat mass from a diet associated with later adiposity because of high fatness at baseline, fat mass at baseline should be used as a covariate. As fat mass was not assessed at baseline (10 years) when data on diet was collected, overweight status at baseline, an indicator of fatness, will be used as a covariate in this paper instead. At focus clinics at age 10 and 13 y height was measured to an accuracy of 0.1 cm using a Harpenden stadiometer (Holtain Ltd., Crymmych, Pembs, UK) and weight was measured to 0.1 kg using the Tanita (West Drayton, Middlesex, UK) TBF 305 body fat analyser weighing scale. BMI was calculated by dividing weight (kg) by height (m) squared and overweight was defined using international age and sex specific cut offs [33].

Puberty is related to significant changes in body composition and the higher energy demands of growth during puberty may be met by consuming a more energy dense diet, therefore puberty was considered as a covariate in this analysis. Puberty (Tanner stage) was reported by parents on behalf of their children in questionnaires sent at age 13 y. Using line drawing parents chose the stage (1 to 5) of development their child had reached. The highest of two ratings (breast or pubic hair for girls; genital or pubic hair for boys) was used to indicate pubertal stage.

Parental SES has been shown to be related to both childhood weight status and diet in ALSPAC [34], [35]. Maternal education was self-reported via questionnaires sent at 32 weeks gestation and was categorised as none, certificate of secondary education, vocational, O level, A level or degree.

TV watching is associated with diet as well as obesity in ALSPAC [36], [37] and was used as a covariate in this paper. Time spent watching TV was reported by parents in questionnaires sent out at age 8 y using the two part question *How much time on average does your child spend watching TV each day (i) on a weekday (ii) on a weekend day* with the options for responses being *Not at all*; *less than 1 hour*; *1–2 hours*; *3 or more hours*. Average daily TV watching was calculated for week days and weekend days combined and categorised as less than 1 or 1–2 or >2 hours/day.

PA is inherently linked to diet and body size via its effect on energy balance. Physical activity was measured over 7 days at age 11.7±0.23 y using MTI actigraph AM7164 2.2 accelerometers (Actigraph, http://theactigraph.com). Data from children that had worn the actigraph for at least 3 days for at least10 hours per day were considered valid. Total physical activity was measured as average counts per minute (cpm) [37], [38].

Dietary variables including energy, fat and fibre intake are on the causal pathway from DED to greater fat mass, therefore these dietary factors were not considered confounders in the analysis. Similarly, parental BMI is related to both childhood fatness and diet but lies on the causal pathway from *FTO* to fatness, as such parental BMI was not considered a confounder in this paper.

### Statistical analyses

Variables were described using mean and standard deviation. Associations between 2 continuous variables were assessed using Pearson's correlation coefficient. T tests or ANOVA were used to test for differences in means in 2 or more groups. Nonparametric tests Mann Whitney or Kruskall Wallis were used when the assumptions of the t test or ANOVA were not met. χ^2^ test was used to assess association between 2 categorical variables or departure of allele frequencies from Hardy Weinberg Equilibrium (HWE). Multiple linear regression analysis was used to model the determinants of fat mass at age 13 y.

Fat mass and FMI were log transformed to approximate the normal distribution. Statistics for log fat mass and log FMI were back transformed for presentation in tables and figures. FMI was used to describe differences between groups in figures and tables so that variation in fatness could be compared independent of height. Fat mass was used as the outcome in regression analyses with height at age 13 y included as a covariate to adjust for body size, which allowed a more meaningful interpretation compared to FMI.

DED (kJ/g) at age 10 y and *FTO* were the primary exposures of interest. A categorical misreporting variable (under-, plausible-, or over-reporter) was included in all analyses of DED as it has been shown to be an important confounder in diet-obesity associations [19]. Basic and adjusted regression models were run. Weight status at 10 y may confound the relationship between DED at 10 y and fat mass at 13 y but may lie on the causal pathway of *FTO* to fat mass at 13 y. Therefore weight status was entered separately into the adjusted model so that its effect on the relationships between DED and fat mass and *FTO* and fat mass could be observed.

Basic models were repeated in a restricted sample with complete data on all variables (n = 2275), in order to check if changes in effect size were a result of inclusion of confounders or a reduction in sample size. An interaction term was calculated by multiplying DED by *FTO* (AA = 3, AT = 2, TT = 1) for each child. This interaction term was then included in a regression model along side both DED and *FTO*. If the β value for the interaction term was different to 0 this was taken as evidence of an interaction. All analyses were completed using SPSS version 14.0 (SPSS Inc, Chicago, IL).

### Power

Prior to the analysis a power calculation was performed using the program Quanto (http://hydra.usc.edu/gxe/) to estimate the size of the smallest interaction effect detectable in a sample of n = 4318 (those with complete data on genotype, diet and fat mass) with a power of 0.8. We assumed a risk allele frequency of 0.39, an additive mode of inheritance, and mean (SD) fat mass, dietary energy density and main effect sizes of DED and the *FTO* gene were estimated from previous data collected in ALSPAC [19], [20]. The smallest interaction effect detectable in a sample of 4318 children was β = 0.3.

## Results

**Figure 2:**
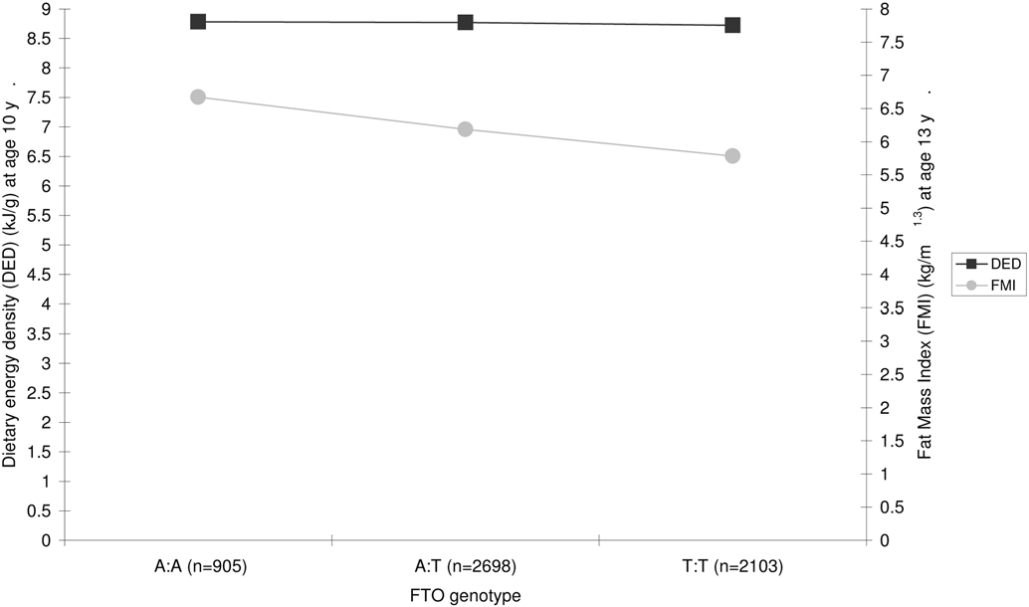
Dietary energy density (kJ/g) and FMI (kg/m^1.3^) by *FTO* genotype in ALSPAC children. Values are means among children with valid data on DED, FTO and FMI (n = 4318).

The minor allele frequency of *FTO* was 0.40 and did not depart from HWE (p = 0.5). FMI at age 13 y was higher among carriers of the high risk A allele (**figure 2**). In a basic regression model, adjusting for height and sex, each A allele was associated with 0.68±0.25 kg more fat mass at 13 y (**table 1**).

**Table 1:** Predicting fat mass at age 13 y from DED at 10 y and *FTO* in all children with data.

Variable	Model	n	β^1^	(95% CI)
DED	A^2^	5527	0.21	(0.12, 0.30)
*FTO*	B^3^	4638	0.68	(0.44, 0.93)
DED	C^4^	4318	0.23	(0.13, 0.33)
*FTO*	C^4^	4318	0.71	(0.47, 0.95)

1 β represents the change in fat mass (kg) for either each kJ/g of DED or each A allele of the *FTO* genotype. Values calculated by back-transforming β from the regression models with log fat mass as the outcome.

2 Model includes height at age 13 y, sex, DED, misreporting of EI.

3 Model includes height at age 13 y, sex, *FTO*.

4 Model includes height at age 13 y, sex, *FTO*, DED, misreporting of EI.

Mean (SD) DED in all children with data on diet at age 10 y was 8.76±1.63 kJ/g (**table 2**). Increased DED at 10 y was associated with a higher EI (r = 0.22, p<0.0001). At age 10 y 63% of children had plausible-reports of EI. Under-reporters of EI at age 10 y were much more likely than plausible-reporters to be defined as overweight at age 10 (42% vs 12%) and 13 y (47% and 19%). Mean (SD) DED was lower among under-reporters compared to plausible-reporters (8.45±1.67 kJ/g vs. 8.87±1.56 kJ/g; p<0.0001). These opposing trends between misreporting and DED and weight status may bias the relationship between DED and fatness towards the null if not accounted for in the analysis. In a basic regression model, adjusting for height and sex, there was no evidence of an association between fat mass at age 13 y and DED at age 10 y (β = 0.01, p = 0.91). However, after adjustment for reporting accuracy each kJ/g of DED at age 10 y was associated with 0.21±0.05 kg more fat mass at age 13 y (**table 1**).

**Table 2:** Dietary energy density (DED), fat mass index (FMI) and allele frequencies of *FTO* in ALSPAC.

	Children with data on DED^4^ or FMI^5^ or *FTO* 6	Children with data on DED and FMI and *FTO* 7	Children with data on DED and FMI and *FTO* and confounders^8^
DED (kJ/g)^1^	8.76 (1.63)	8.70 (1.59)	8.64 (1.52)
FMI (kg/m^1.3^)^2^	6.16 (1.81)	6.09 (1.80)	6.16 (1.78)
*FTO* 3	0.395	0.396	0.401

1 Data are mean (SD).

2 Data are geometric mean (SD) of Log FMI.

3 Data are minor allele frequencies.

4 n = 7430.

5 n = 6023.

6 n = 8893.

7 n = 4318.

8 n = 2275.

**Figure 3:**
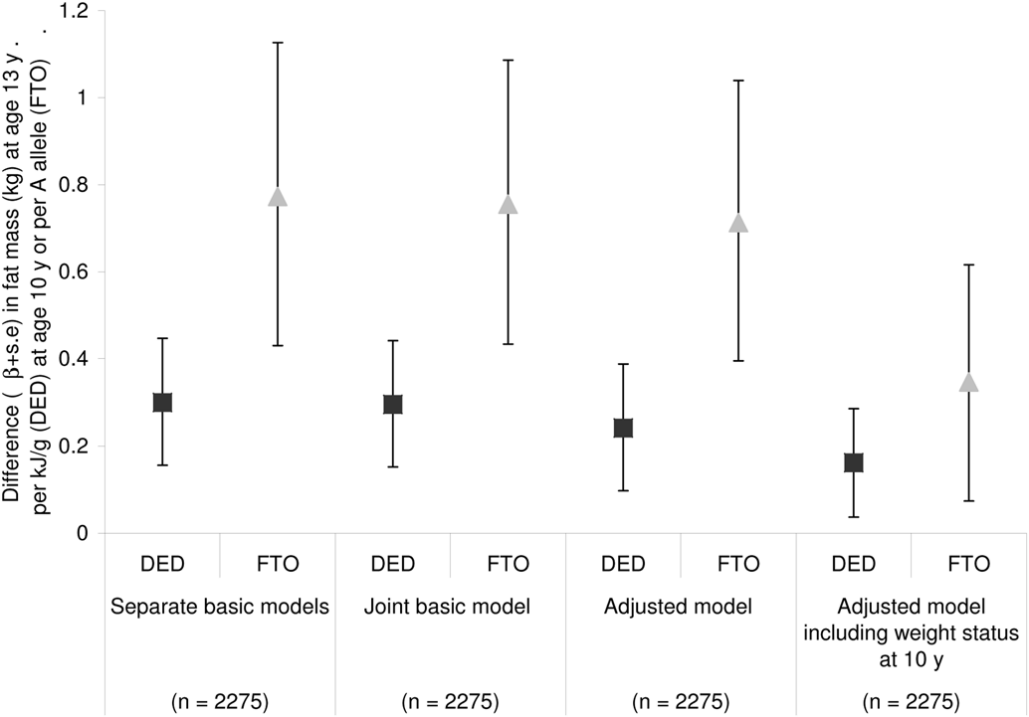
Predicting fat mass at age 13 y from DED at 10 y and *FTO* in children with complete data (n = 2275). Values are regression coefficients and 95% confidence intervals. A statistically significant effect is indicated by a 95% CI that does not include 0. Separate basic models contain either FTO or DED and are adjusted for height at age 13 y and sex; misreporting of EI is included for models with DED. Joint basic model contains FTO and DED and is adjusted for height at age 13 y, sex and misreporting of EI. Adjusted model includes FTO and DED adjusted for height at age 13 y, sex, puberty (Tanner stage 1 to 5) at 13 y, misreporting of EI (under-, plausible-, or over-reporter), EI from drinks (kJ/day) at 10 y, maternal education (none, certificate of secondary education, vocational, O level, A level or degree), TV watching at 8 y (less than 1/1–2/2 hours/day), physical activity at 11 y (cpm). Adjusted model including overweight status at 10 y contains the same variables as Adjusted model in addition to overweight status at 10 y (defined by International Obesity Task Force criteria [34]).

Carriers of the A allele had a higher EI at 10 y [24], but there was no evidence of an association between *FTO* and DED at age 10 y (**figure 2**). There was no evidence of interaction between *FTO* and DED at 10 y on fat mass at 13 y (β = 0.005, p = 0.51). Multiple regression analysis containing both DED and *FTO* in the same model showed each factor had independent effects on fat mass at 13 y (**table 1**). When the analyses were repeated in the sample with complete data (n = 2275) there was no change in the size of the effect estimate for either *FTO* or DED at 10 y when they were both included in the model (**figure 3**). Furthermore, the inclusion of potential confounders in the model had little impact on the effect estimates (**figure 3**). In the adjusted model each A allele was associated with 0.71±0.16 kg more fat mass at 13 y and each kJ/g was associated with 0.24±0.07 kg more fat mass at 13 y (**figure 3**). The addition of weight status at 10 y to the adjusted model attenuated the effect on fat mass at 13 y for both *FTO* and DED so that each A allele was associated with 0.35±0.13 kg more fat mass at 13 y and each kJ/g was associated with 0.16±0.06 kg more fat mass at 13 y.

The impact of missing data was analysed. Children who attended clinics were more likely to come from more affluent or better-educated families compared to those that did not attend (data not shown). DED was lower in those children with complete data on all confounders compared to those children with missing data (p<0.001) (**table 2**). There was no evidence of a difference in FMI (p = 0.82) or allele frequency (p = 0.63) among those children with complete data compared to those with missing data (**table 2**).

## Discussion

In this prospective analysis of the interaction between DED and *FTO* we have found no evidence of interaction. The results presented suggest that DED at age 10 y and *FTO* contribute independently to greater fat mass at age 13 y. Each A allele of *FTO* was associated with 0.35 kg more fat mass at 13 y and each 1 kJ/g DED at 10 y was associated with 0.16 kg more fat mass at age 13 y, after controlling for misreporting of energy intake, gender, puberty, maternal education, TV watching, physical activity and overweight status at 10 y. To our knowledge this is the first study of the effect of *FTO* on modifying the impact of DED on fatness and these results are an important addition to our understanding of the multi-factorial origins of obesity.

No previous study has found a linear relationship between DED and fat mass in children, therefore comparing the effect size with the existing literature is not possible. Data from a sub-sample of ALSPAC found that DED at 7 y was related to excess adiposity at 9 y but not fat mass across the whole range [19]. Furthermore there was no evidence of a relationship between DED at 5 y and excess adiposity or fat mass at 9 y. The changing effect of DED on later fat mass with age suggests over-consumption in response to energy dense foods may become more likely as innate appetite control becomes more susceptible as children get older to disruption by external cues such as high palatability.

The addition of weight status at 10 y to the regression models attenuated the effects of both *FTO* and DED at 10 y on fat mass at 13 y. For DED this suggests that to some extent overweight children are more likely to consume more energy dense diets, which explains some of the impact of DED at 10 y on fat mass 3 years later. However, given that the effect of DED at 10 y remains (all be it reduced in size) this supports a causal role for DED in increasing fat mass over time. For *FTO* the inclusion of weight status at 10 y explained part of the effect on fat mass at 13 y, which suggests that the whole effect of *FTO* on fatness at a given time point, is the accumulation of many small effects over time.

The present analysis provides no evidence that the effect of DED on fat mass is altered by the *FTO* genotype. This lack of evidence could indicate that the A allele of the *FTO* gene does not enhance the effect of DED on fat mass or it could suggest that the real interaction effect is smaller than 0.3 (the smallest effect detectable with our sample size). There is also no evidence that the effect of *FTO* is explained by changes in DED as there was no change in the effect estimate for *FTO* when DED was included in a multiple regression. This reinforces a previous analysis of ALSPAC data [24] suggesting that *FTO* increases the consumption of all foods and not specifically highly palatable, energy-dense foods. The evidence presented here suggests that DED and *FTO* simply combine to increase the risk of obesity in an additive manner.

The public health importance of the present findings is that the effect of the high risk A allele of the *FTO* genotype on fat mass may be, in part, offset by dietary changes to lower DED. Intervention studies have shown that free-living adults can feasibly reduce, for up to 12 months, their DED by at least 2 kJ/g via the replacement of high fat with low-fat foods and the inclusion of more fruits and vegetables in their diet [39], [40]. If a similar reduction in DED could be achieved in children then, based on the effect estimate in the present analysis, this may lead to a third of a kilogram reduction in fat mass. This potential effect of lowering DED could balance out some of the extra fat mass associated with carrying an A allele of *FTO*.

There are several strengths to this study. The data analysed come from a large population based cohort, which has been followed prospectively for 13 y with detailed records of food intake, direct measures of body composition and good measures of a range of potential confounders. Previous work has shown that the diet observed in this cohort is comparable to that observed in nationally representative samples of young UK children [41], as was the observed prevalence of overweight and obesity [42]. As with all observational studies, it is possible that the association between DED and fat mass was the result of some unmeasured confounding factor. A weakness of the study is that complete dietary, genetic and body composition data was available for only 30% of the original sample. Comparison of data among children with and without complete data showed no difference in fatness and allele frequency. However, children with complete data did have a lower DED compared to those without complete data, which may mean that the effect of DED on fatness has been under estimated.

This study reveals the multi-factorial origin of obesity and indicates that although the *FTO* gene may put some children at greater risk of obesity, encouraging a low dietary energy density may be an effective strategy to help all children avoid excessive fat gain.
